# Case Report: Application of *ex-vivo* drug sensitivity testing to identify personalized treatment options for an adolescent with diffuse midline glioma

**DOI:** 10.3389/fonc.2025.1606575

**Published:** 2025-08-07

**Authors:** Philip K. Tan, Timothy J. Martins, Pamela S. Becker, Robert J. Wechsler-Reya, John Ross Crawford

**Affiliations:** ^1^ The Cure Starts Now Foundation, Cincinnati, OH, United States; ^2^ Yuvaan Tiwari Foundation, Atlanta, GA, United States; ^3^ Institute for Stem Cell and Regenerative Medicine, University of Washington School of Medicine, Seattle, WA, United States; ^4^ Department of Hematology and Hematopoietic Cell Transplantation, City of Hope National Medical Center, Duarte, CA, United States; ^5^ Department of Hematologic Malignancies Translational Science, City of Hope National Medical Center, Duarte, WA, United States; ^6^ Department of Medicine, University of Washington, Seattle, WA, United States; ^7^ Cancer Genome and Epigenetics Program, National Cancer Institute (NCI)-Designated Cancer Center, Sanford Burnham Prebys Medical Discovery Institute, La Jolla, CA, United States; ^8^ Department of Neurology and Herbert Irving Comprehensive Cancer Center, Columbia University Medical Center, New York, NY, United States; ^9^ Rady Children’s Hospital, San Diego, CA, United States; ^10^ Children’s Hospital of Orange County, Orange, CA, United States; ^11^ Department of Pediatrics and Neurology, University of California Irvine, Irvine, CA, United States

**Keywords:** diffuse midline glioma, functional precision medicine, drug sensitivity testing, molecular guided therapy, pediatric brain cancer

## Abstract

Diffuse midline glioma (DMG) is a pediatric brain cancer that has a dismal prognosis with limited treatment options. We present the treatment course and outcome of an adolescent male diagnosed with a thalamic DMG carrying a histone H3.3 K27M (H3K27M) alteration. Tumor biopsies were taken at diagnosis for histological analysis, molecular profiling, and *ex vivo* drug sensitivity testing (DST). Seven months after diagnosis, the patient had recurrent/progressive disease after radiotherapy and an ineffective molecular-guided therapy based on tumor molecular profiling. The patient then started a novel functional precision medicine (FPM)-guided two-drug combination of disulfiram, based on the DST results of this drug on the patient’s tumor cells obtained at diagnosis, and ONC 201, the only drug that has advanced to a phase III clinical trial for H3K27M-DMG. Neuroimaging demonstrated a treatment response, and the patient lived for fifteen months after starting this personalized therapy. Disulfiram was discontinued after three months due to significant peripheral neuropathy. Our case describes the feasibility and limitations of using DST of patient-derived tumor cells to identify potentially effective personalized and novel therapies for DMG, which should be evaluated for efficacy and safety in formal N-of-1 clinical trials settings. We discuss the benefits and risks of this approach, particularly considering its use in children, adolescents, and young adults with pediatric brain cancers.

## Introduction

Diffuse midline glioma (DMG) is a high-grade glioma that occurs mainly in children and more rarely in adults. It is the deadliest brain cancer in children and young adults with a life expectancy of about a year after diagnosis (1), although adults have a significantly longer life expectancy (2, 3). DMG is molecularly defined by a lysine-27 to methionine mutation in histone H3 (H3K27M) that leads to global hypomethylation of this residue and consequent epigenetic changes in gene expression patterns that are likely to promote oncogenesis (4, 5). The tumors occur within deep midline regions of the brain and spinal cord and are infiltrative in nature such that surgical resection is risky and often ineffective. There are no FDA-approved therapies and patient outcomes have not improved over decades. Radiation is the only standard of care therapy and is merely palliative, and clinical trials of chemotherapeutic and targeted agents after radiotherapy have been largely ineffective. Upon tumor progression, DMG patients have even fewer treatment options and a median life expectancy of only three months if no further treatments are pursued (6, 7).

Functional precision medicine (FPM) is a promising approach that utilizes drug sensitivity testing (DST) of patient-derived tumor cells either alone or in combination with molecular profiling as part of multi-omics testing to predict effective individualized treatment options (8–13). However, DST has not been widely applied to children and adolescents with pediatric brain cancer. This case describes an adolescent DMG patient whose tumor cells were subjected to *ex vivo* DST at diagnosis. Seven months later at the time of tumor progression, the patient started a personalized and experimental two-drug combination therapy of disulfiram, based on the DST results of this drug, and ONC201 (dordaviprone), the only drug that has advanced to a phase III clinical trial for H3K27M-DMG (14). This FPM-based therapy led to a tumor regression and the patient survived for 15 months after starting this treatment and for 22 months after diagnosis, although it also induced a peripheral neuropathy that reduced his quality of life and led to discontinuation of therapy. Our case highlights the challenges and opportunities of DST and serves as a basis for potential FPM-based clinical trials for patients with DMG.

## Case description

**Figure 1:**
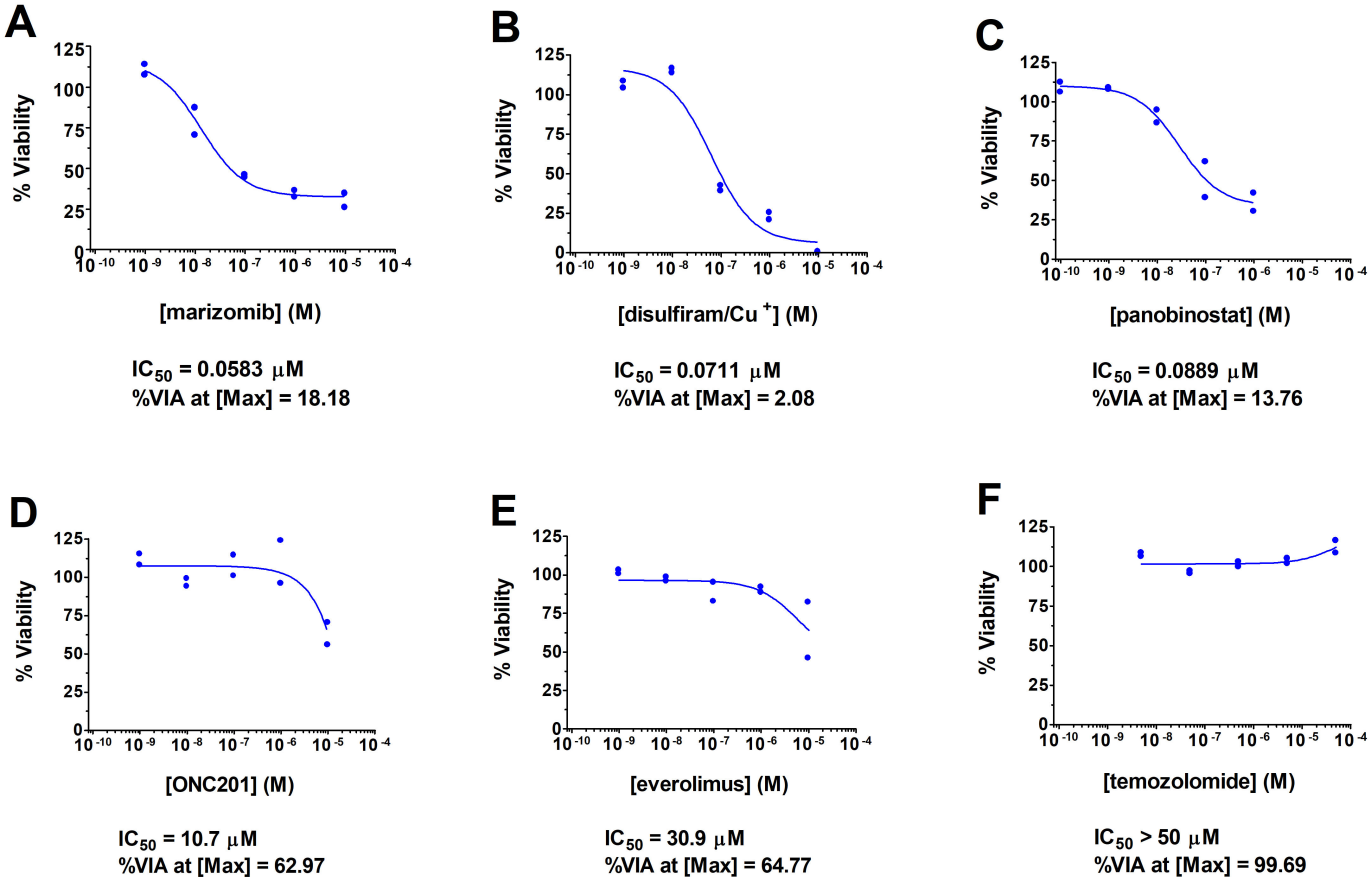
Summary of DST results for drugs used by the patient and for marizomib. DST curves are shown for marizomib **(A)**, two other drugs with high potency that the patient used [disulfiram **(B)** and panobinostat **(C)**], and for three drugs with low potency that the patient used [ONC201 **(D)**, everolimus **(E)**, and temozolomide **(F)**]. Briefly, the freshly biopsied tumor cells were dispersed by enzymatic digestion, shipped on ice to a CLIA laboratory at the University of Washington (16), and on the next day were plated with each drug on opaque 6x384-well plates at 2000 cells per well. After a 3-day incubation, cell viability was measured using CellTiter Glo 2.0 (Promega Corp., Madison, WI, USA). Each drug was assayed at five concentrations in duplicate. XLFit from IBDS was used to generate dose-response curves using the 4-parameter logistic dose response model, tumor cell killing potency of each drug at the half-maximal concentration in micromolar, or IC_50_, and percentage of viable cells at the maximal concentration of each drug (%VIA at [Max]) as a measure of drug efficacy; the drug potency and efficacy values are shown below each dose response curve. Cu^+^, copper in the form of copper gluconate.

An adolescent male presented with a 1-month history of fatigue and intractable vomiting. Examination was significant for obtundation, and right hemiparesis and neuroimaging revealed a left thalamic neoplasm with obstructive hydrocephalus and herniation. He underwent emergent external ventricular drain placement, fenestration of the septum pellucidum, and tumor biopsy. Histological staining and molecular analysis of the biopsies revealed the presence of the *H3F3A* K27M mutation along with mutations in *TP53* (with accompanying loss of heterozygosity), *PIK3CA*, and a *SMARCE1* mutation that was sub clonal (**Table 1**). Separately, the biopsied tumor cells were subjected to a feasibility study featuring multi-omics analysis, DNA methylation profiling, immunogenic potential analysis, and *ex vivo* DST with a customized panel of 175 FDA approved and investigational cancer drugs (15; John R. Crawford and Robert Wechsler-Reya, manuscript in preparation) at a Clinical Laboratory Improvement Amendments (CLIA)-certified laboratory at the University of Washington (16). Consistent with previously reported DST results on patient-derived DMG tumor cells, drugs with the most potency in viability assays included proteasome inhibitors such as marizomib and histone deacetylase (HDAC) inhibitors such as panobinostat (17, 18). Disulfiram also caused significant lethality of tumor cells, with an IC_50_ of 71 nanomolar and a tumor cell viability of just 2% at the maximal dose. **Figure 1** shows the DST results of marizomib, as well as the results for other drugs that the patient used with his treatment plan. The **Supplementary Table** lists the *ex vivo* DST results for all the drugs that were evaluated.

**Table 1:** Tumor molecular analysis, somatic alterations.

Gene/Protein Variant	Classification	Mutant Allele Frequency
*H3F3A*/H3.3-K27M (H3K27M)	Pathogenic	31%
*PIK3CA*/PI3K-E545G	Pathogenic	44%
*TP53*/p53-D281G	Pathogenic	72%
*SMARCE1*/BAF57-E156*	Possibly Pathogenic	10% (subclonal)

Variants are listed as the gene name followed by the protein name and amino acid alteration. For *TP53*, there was an accompanying loss of the other allele due to copy-neutral loss of heterozygosity of chromosome 17. “*” indicates that a stop codon was produced by the mutation leading to truncation of the protein.

**Figure 2:**
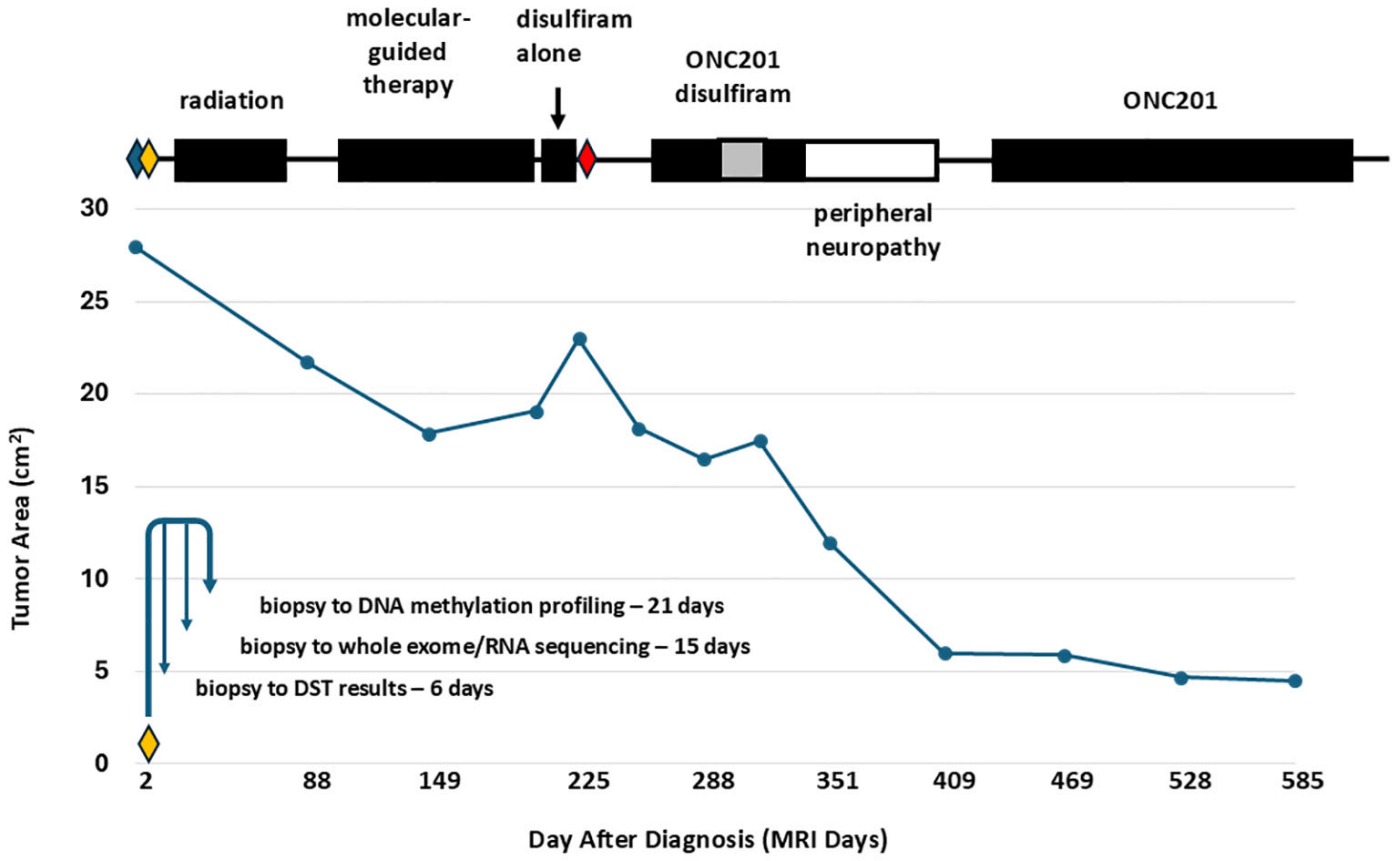
Treatment course and thalamic DMG tumor area measurements showing tumor regression with radiation therapy and with the FPM therapy of disulfiram and ONC201. The graph plots the measured area of the primary thalamic DMG tumor from each MRI. Above the graph, the time periods for the different treatments are shown in black boxes. Disulfiram was used at 250 mg QD, except during a time period at 250 mg BID (gray box). Surgeries are indicated by diamonds: blue diamond, external shunt placement and septostomy (day 1); yellow diamond, external shunt removal and third ventriculostomy (day 7); red diamond, internal shunt placement and septostomy (day 226). The lower left of the graph shows a timeline from tumor biopsy on the day 7 surgery to profiling including DST. The time period of the peripheral neuropathy is indicated as an open box. Standard of care radiation therapy led to tumor regression, observed as a reduction in tumor area on days 88 and 149. Following radiation therapy, an ineffective molecular-guided therapy with the drug combination temozolomide, panobinostat, everolimus, and hydroxychloroquine led to tumor progression, observed as an increase in tumor area on days 203 and 225. The FPM therapy of disulfiram alone, from days 210-224, disulfiram and ONC201, from days 258-333, and ONC201 alone led to a dramatic decrease in tumor area. The patient died on day 677 from treatment-resistant secondary tumors that were first visualized on day 255.

**Figure 3:**
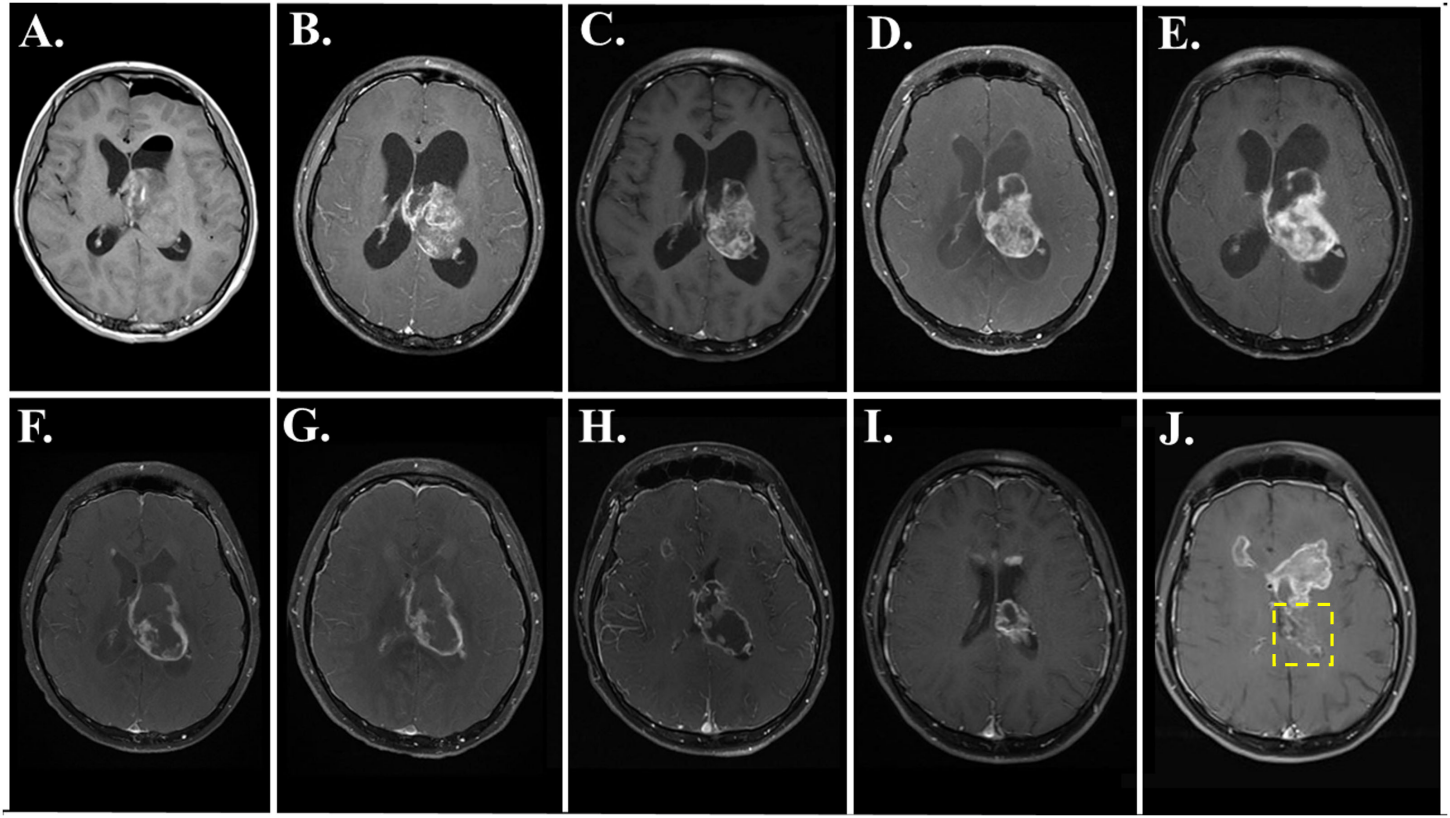
MRI images showing primary DMG tumor regression during and after FPM therapy with disulfiram and ONC201. T1-post gadolinium axial images are shown from the following days: **(A)** day 2; **(B)** day 88; **(C)** day 149; **(D)** day 203; **(E)** day 225; **(F)** day 255; **(G)** day 288; **(H)** day 351; **(I)** day 409; and **(J)** day 469. In J, the location of the primary tumor is within the yellow box, and secondary tumors are evident above the location of the primary tumor.

**Figure 2** shows the course of treatment for the patient along with a graph of the area of the primary thalamic tumor for each magnetic resonance imaging (MRI), and the timing of molecular profiling and DST (lower left). **Figure 3** presents representative post-gadolinium T1-weighted MRI images of the primary thalamic DMG tumor, shown in **Figure 3A** at diagnosis. Standard of care radiation therapy (proton therapy of 60 Gy over 30 cycles) from days 22–63 led to a continual reduction in tumor area based on imaging on days 88 and 149 (**Figures 2**, **3B, C**). While in radiotherapy, the patient and his family expressed an interest in drug combination therapies, and since there are no FDA-approved therapies for DMG they were informed of the benefits and risks of using novel drug combinations, and they provided consent to use a molecular-guided combination drug therapy based on tumor molecular profiling obtained at diagnosis. On day 102 the patient started an individualized therapy consisting of panobinostat, everolimus, temozolomide, and hydroxychloroquine. An MRI on day 203 showed tumor progression (**Figure 3D**) and hence this combination therapy was deemed ineffective and was discontinued after 3.5 months.

Based on the success of a feasibility study (15), the *ex vivo* DST results of the patient’s tumor cells were reviewed for the next treatment options as a salvage therapy, and the patient and family consented to a novel drug combination and FPM-based therapy of disulfiram and ONC201. Disulfiram, an FDA-approved drug for treating alcoholism that is in evaluation as a repurposed drug for treating high grade glioma and other cancers (19), resulted in high potency cell death in *ex vivo* DST (**Figure 1**). Meanwhile, ONC201 was at that time was available in Germany for off-trial use and serves as a potential molecular profiling therapy for DMG tumors carrying the H3K27M mutation (20, 21). Seven months after diagnosis on day 210, disulfiram was initiated alone at 250 milligrams (mg) once per day (QD). After 15 days, the disulfiram treatment was interrupted due to worsening hydrocephalus and an MRI on day 225 showed further tumor progression (**Figure 3E**). The patient then underwent another surgery for internal shunt placement, and did not receive any cancer therapies for over a month while in recovery. An MRI on day 255 at the end of this recovery period (**Figure 3F**) showed reduced hydrocephalus, and interestingly tumor regression from the previous month.

The patient then received the combination of disulfiram and German-sourced ONC201 starting on day 258. ONC201 was administered once per week at 680 mg, corresponding to 8.5 mg per kilogram of body weight. Disulfiram was initially administered at 250 mg QD for the first month, then increased to 250 mg twice per day (BID) for one month consistent with the dosing used in clinical trials for glioblastoma (22, 23) and then decreased to 250 mg QD for another 18 days. The disulfiram-ONC201 combination therapy was stopped on day 333 due to significant painful peripheral neuropathy. During this combination therapy, the tumor regressed continually month by month based on MRIs on days 288 (**Figure 3G**), and 351 (**Figure 3H**). After this therapy was stopped, MRIs on day 409 (**Figure 3I**) and day 469 (**Figure 3J**), with the primary tumor indicated within the yellow box) showed even further tumor regression.

The disulfiram-ONC201 combination therapy was stopped due to the onset of peripheral neuropathy featuring intense neuropathic pain in the feet and distal motor weakness/areflexia which led to inability to ambulate and subsequent hospitalization. MRI spine did not show any evidence of nerve root enhancement or cord signal abnormality. A lumbar puncture to assess for albuminocytologic dissociation was recommended but declined and the patient was treated for symptomatic neuropathic pain management and physical therapy. The painful neuropathy resolved after three months; however, the patient never regained his ability to ambulate due to persistent bilateral foot drop. After resolution of the neuropathy on day 433, the patient resumed ONC201 alone without disulfiram, and the peripheral neuropathy did not recur. Tumor progression appeared originating from the frontal horns of the right and left ventricles (first noticeable on day 225, **Figure 3F**, and prominent on day 469, **Figure 3J**) and the left cerebellar peduncle. The primary thalamic tumor never recurred, and the patient died of disease on day 677.

## Discussion

Our case study demonstrates feasibility of *ex vivo* DST of the tumor cells from a DMG patient obtained at diagnosis, and a favorable treatment response of a primary thalamic DMG and extended survival of the patient to an off-trial N-of-1 FPM salvage therapy, consisting of a two-drug combination of disulfiram, a repurposed cancer drug (19) that was selected based on the DST results, and ONC201, an investigational drug for H3K27M-DMG currently being evaluated in phase III clinical trial setting (14). The patient started this therapy seven months after diagnosis, after radiotherapy and an ineffective molecular-guided therapy, when the tumor was recurrent and when the patient needed surgery for worsening hydrocephalus. Given the lack of clinical trial options available and at the request of the patient and family with informed consent, a novel personalized combination salvage therapy approach based on the DST results obtained at diagnosis was undertaken. After starting the FPM-guided therapy, the primary tumor regressed quickly and continuously in the following months, never recurred, and the patient survived for another fifteen months. This survival time compares favorably to recurrent DMG patients that lived for only three months when no further treatments were pursued or for 5–6 months after re-irradiation (6, 7). However, most DMG patients in survival studies were in children 18 years of age or younger. It is possible that the longer survival time of this adolescent is related to age-dependent biological differences between pediatric and adult DMG which may be responsible for improved survival reported in adults (2, 3). Ultimately, the treatment response in this case was mixed as secondary tumors in other areas of the brain that were initially visualized during this FPM therapy were treatment-resistant resulting in tumor progression and eventual death.

The FPM therapy was discontinued after three months due to peripheral neuropathy that was likely caused by disulfiram (24–28). Disulfiram-induced neuropathy is rare and dose dependent, and has been documented in clinical trials of glioblastoma patients where it was dosed at 500 mg QD or higher (22, 23), similar to the dosing used by this patient when the neuropathy first appeared. Disulfiram was shown to be safe in adolescents at a lower dose of 200 mg daily (29), and no studies have been reported in children. The combination of disulfiram and ONC201 has not been investigated, and therefore the mechanisms for the neuropathy due to a drug-drug interaction can only be speculative. The adverse event in this adolescent patient highlights the safety concerns of using uncharacterized drug combinations and treating adolescent and pediatric brain cancer patients with drugs that were developed for other indications in adults. After the neuropathy subsided, the patient declined to restart disulfiram at a lower dose due to the severity of neuropathic side effects. The family and patient also did not seek treatment using other drugs based on the DST results due to uncertainties with dosing for off-label use in an adolescent with DMG. Instead, they focused on treating the patient with ONC201 alone which does not cause peripheral neuropathy (30, 31), along with other palliative therapies.

*Ex vivo* DST of the patient’s tumor cells was part of a feasibility study for pediatric brain cancer patients that also included several multi-omics tumor profiling methods. Reliable DST results were obtained within one week, and all tumor profiling results were obtained within an average time of three weeks after tumor biopsy (15, **Figure 2**, lower left). Subsequently, these profiling methods have been used in an FPM clinical trial for patients with recurrent medulloblastoma (NCT05057702, PNOC027). Our patient’s tumor cells, in contrast to this clinical trial, were profiled just after diagnosis, and the DST results were not used to inform the molecular-guided therapy that the patient tried after surgery and standard of care radiotherapy. Therefore, it is important to note that the FPM therapy for this patient was initiated seven months after DST of the patient’s biopsied tumor cells and after prior therapies. Due to extensive tumor heterogeneity and adaptive resistance of high-grade gliomas, it is likely that time and prior therapies led to molecular changes in the tumor and hence resistance of the secondary tumors to the FPM therapy (32–36). Ideally, FPM therapy should be initiated immediately after DST testing, when the personalized therapy would likely be a better match with the DST results. The option of obtaining additional tissue for DST at the time of progression was not performed based upon patient/family and provider decision.

One must interpret the favorable treatment response with caution as we cannot know whether it was due to disulfiram, ONC201, or the drug combination. ONC201 has demonstrated promising early-stage clinical results including durable responses in a few individuals (30, 37–41). Disulfiram is approved for alcohol abuse and has been repurposed as a cancer therapy due to broad-spectrum anticancer activities (19), and has preclinical efficacy against DMG (42, 43). The efficacy of disulfiram on patients with DMG, however, is unknown, and its use on DMG patients has not been previously reported. The patient initially used disulfiram alone for only 15 days, and the subsequent MRI just 30 days later showed tumor regression (**Figure 2**, and compare **Figures 3E, F**), suggesting a favorable treatment response to disulfiram. In clinical trials, disulfiram demonstrated efficacy in a phase IIb trial for non-small cell lung cancer (44). However, there was no efficacy in a recent clinical trial for glioblastoma (45), The combination of disulfiram and ONC201 on the efficacy of any cancer, including potential synergistic effects, is unknown. Notably, in the absence of other treatment options, the patient and his family were willing to explore using ONC201 in a novel combination with another available and off-trial drug based on functional DST of the patient’s tumor cells.

Another limitation of this case report is extrapolating *ex vivo* DST results to *in vivo* drug efficacy. In this regard, DMG is challenging because there are no known effective individual or combination drug therapies, and drugs used for treating DMG also need to be brain penetrant. A starting point is using the drug potencies from *ex vivo* DST as a benchmark for potentially efficacious drug exposure. For example, *ex vivo* disulfiram had a high killing potency (IC_50_) of 0.071 μM against the patient’s tumor cells (**Figure 1**), and steady state plasma concentrations of disulfiram in patients reach levels that are up to ten times higher (46), suggesting that efficacious concentrations disulfiram could be achieved for this patient. Any brain penetrant drug with these features could be a candidate for an FPM therapy, and disulfiram was selected as a personalized therapy in part due to clinical trials in adult glioma that were ongoing at the time. However, there are other *in vivo* drug parameters to consider for potential efficacy. Disulfiram may have unfavorable metabolism and bioavailability that limits its efficacy (46, 47), perhaps explaining why disulfiram has failed in clinical trials for glioblastoma (23, 45). Meanwhile, ONC201 had an IC_50_ of around 10 μM against the patient’s tumor cells (**Figure 1**). Peak plasma concentrations of ONC201 reached a mean concentration of 12.2 μM when dosed in patients on two consecutive days per week (48). However, peak plasma concentrations were lower than 10 μM in most patients that were dosed once per week (31), as ONC201 was dosed for this patient, suggesting that this patient’s tumor may have been resistant to pharmacologically relevant concentrations of ONC201 and consistent with the lack of efficacy of ONC201 in clinical trials for some patients (38). However, ONC201 may have a higher anti-tumor efficacy *in vivo* through modulation of interactions between tumor cells with the nervous and immune systems (49, 50), interactions that may not occur in *ex vivo* DST. Therefore, when interpreting DST results for potential *in vivo* efficacy, it is important to understand the pharmacokinetics and mechanism of action of each drug of interest, as well as level of brain penetrance for treating brain cancers such as DMG.

Prior to the FPM therapy, the patient used an individualized molecular-guided therapy based on tumor genetic profiling (**Table 1**) consisting of temozolomide, panobinostat, everolimus, and hydroxychloroquine. At the time, everolimus and panobinostat were recommended for treating *PIK3CA*-mutated H3K27M-DMG (20), and temozolomide is a standard of care therapy for adult high-grade glioma (51). Unfortunately, this molecular-guided therapy proved ineffective. If the *ex vivo* DST results were used to inform therapeutic options at that time, they would have encouraged the use of panobinostat due to its high potency for killing the patient’s tumor cells (**Figure 1**) as well as preclinical efficacy in DMG (17, 18). Panobinostat was available at the time by accelerated FDA approval for treating multiple myeloma and was investigated in a phase I clinical trial for DMG (52), but has since been withdrawn due to inability of the provider to conduct a required post marketing trial (53). In contrast, the DST results would have discouraged the use of everolimus and temozolomide due to their low potency and lack of activity, respectively (**Figure 1**). Hydroxychloroquine was not examined in the DST. Meanwhile, tumor DNA methylation profiling that was concurrently done with DST for this patient (15, data not shown) would have also discouraged the use of temozolomide, which revealed an absence of methylation of the promoter for *MGMT*, encoding 6-O-methylguanine-DNA methyltransferase, that predicts temozolomide resistance (54). The *ex vivo* DST and tumor DNA methylation profiling results of this patient’s tumor cells may explain the failure of this molecular-guided therapy.

Another limitation to precision medicine for pediatric oncology patients is that childhood tumors have a 14-fold lower somatic mutation rate and hence fewer actionable mutations than adult cancers (55, 56). For this patient, H3K27M and *TP53* mutations, which occur in 60% of H3K27M-DMG patients, are not directly actionable, while *PI3KCA*, which occurs in 15-20% of all DMG patients (57–59) and encodes phosphatidylinositol 3-kinase (PI3K), was the only actionable mutation. However, none of the FDA-approved PI3K inhibitors are brain-penetrant, and the investigational brain-penetrant inhibitor paxalisib was not available at the time. But even for adult cancer patients in large precision medicine studies, only 10% had beneficial treatment responses to personalized therapies based on tumor molecular profiling (60, 61). Similarly, a trial using molecular-guided therapies on nineteen DMG patients showed no clinical benefit (62). In contrast, certain FPM clinical trials yielded much better results. For example, 92% of patients who received personalized therapies informed by DST achieved stable disease or better (12). Exceptional treatment responses using FPM have also been reported in patients with pediatric cancer (8, 11).

Our case report promotes the wider use of DST for patients with DMG and other childhood brain cancers preferably in a clinical trial setting. For patients with DMG that have no FDA-approved treatment options, DST can be used to identify potentially effective investigational drugs. FPM for such patients should be used in N-of-1 clinical trials to match the results with the most appropriate therapy (63–65). The drugs used in DST should also be optimized for each tumor type. For example, for brain cancers, all brain-penetrant FDA-approved, investigational, and repurposed drugs should be included in the drug screen. Furthermore, various relevant drug combinations should also be evaluated in DST, including drug combinations in current clinical trials (e.g. ONC201 combined with paxalisib for DMG, clinical trial NCT05009992). FPM clinical trials are typically performed for patients with relapsed or progressive disease. However, for DMG patients we also support the use of DST upon initial biopsy or tumor resection, as was done for the patient in this study, to define FPM therapies for newly diagnosed patients. The inclusion of repurposed drugs such as disulfiram is important because these drugs are available and often inexpensive, thereby providing accessible off-label treatment options (66). However, in some cases these may be associated with significant dose limiting side effects as seen in our patient.

An expanded use of DST may provide valuable information that may lead to rapid identification of promising new therapies that would otherwise not be identified through conventional molecular tumor analysis. DST may be an option for pediatric brain tumors such as ependymoma that lack somatic driver mutations or for tumors such as DMG where there is no curative therapy. While our reported case demonstrates the feasibility of *ex vivo* DST in a single patient, many questions remain regarding the generalizability and mechanistic validation of the DST results. *Ex-vivo* testing of drug combinations from patient derived tumor cells is an attractive area of future study, but novel personalized combination therapies informed by *ex vivo* DST may also be associated with significant adverse events as was the case in our patient. DST together with pharmacologic predictive data of CNS penetrability (when available) and drug safety information should be explored further in the clinical trial setting.

## Data Availability

The raw data supporting the conclusions of this article will be made available by the authors, without undue reservation.

## Supplementary material

Supplementary Table 1 Summary of compounds used in the *ex-vivo* drug screening of DMG.

Supplementary Methods High-throughput drug screening (Clinical Laboratory Improvement Amendments–approved assay) The cell populations were analyzed after a 72-hour exposure to 5 customized drug concentrations (within the range of 5 pmol/L to 100 μmol/L) of each drug spanning 5 logs. After exposure viability was determined using CellTiter-Glo luminescent reagent (Promega) per manufacturer’s protocol, then the plates were analyzed with the EnVision Multilabel plate reader (Perkin Elmer). XLFit (IDBS), a Microsoft Excel Add-in, was used to analyze the data and generate dose–response curves based on standard 4- parameter logistic fit [i.e., fit = (A + (B/(1 + ((x/C)^D)))) where A and B equal minimum and maximum asymptotes, C equals IC50 and D equals slope]. The AUC values were calculated using the XLFit software utilizing minimum and maximum concentrations of drugs/compounds within the panel as the limits of the AUC calculation. For each plate, data were normalized to DMSO 100% viability and blank controls. The samples were not frozen but freshly, dissociated to single cell suspensions within hours from surgically removed tumors that were kept on ice prior to shipping. The drug sensitivity assay was initiated within 48 hours of surgery. Clinical Laboratory Improvement Amendments standards require that thawed cell line data be repeated for standardization of the assay every 6 months. The assay results were highly reproducible.
