# Supplementary material for: Case Report: Application of ex-vivo drug sensitivity testing to identify personalized treatment options for an adolescent with diffuse midline glioma
Source: Front Oncol. 2025 Aug 7;15:1606575. doi: 10.3389/fonc.2025.1606575 (PMC12367499; doi:10.3389/fonc.2025.1606575)
Supplement: Supplementary Methods — High-throughput drug screening (Clinical Laboratory Improvement Amendments–approved assay) The cell populations were analyzed after a 72-hour exposure to 5 customized drug concentrations (within the range of 5 pmol/L to 100 μmol/L) of each drug spanning 5 logs. After exposure viability was determined using CellTiter-Glo luminescent reagent (Promega) per manufacturer’s protocol, then the plates were analyzed with the EnVision Multilabel plate reader (Perkin Elmer). XLFit (IDBS), a Microsoft Excel Add-in, was used to analyze the data and generate dose–response curves based on standard 4- parameter logistic fit [i.e., fit = (A + (B/(1 + ((x/C)^D)))) where A and B equal minimum and maximum asymptotes, C equals IC50 and D equals slope]. The AUC values were calculated using the XLFit software utilizing minimum and maximum concentrations of drugs/compounds within the panel as the limits of the AUC calculation. For each plate, data were normalized to DMSO 100% viability and blank controls. The samples were not frozen but freshly, dissociated to single cell suspensions within hours from surgically removed tumors that were kept on ice prior to shipping. The drug sensitivity assay was initiated within 48 hours of surgery. Clinical Laboratory Improvement Amendments standards require that thawed cell line data be repeated for standardization of the assay every 6 months. The assay results were highly reproducible. [file DataSheet1.docx]

Supplementary Methods:

High-throughput drug screening (Clinical Laboratory Improvement Amendments–approved assay) The cell populations were analyzed after a 72-hour exposure to 5 customized drug concentrations (within the range of 5 pmol/L to 100 μmol/L) of each drug spanning 5 logs. After exposure viability was determined using CellTiter-Glo luminescent reagent (Promega) per manufacturer’s protocol, then the plates were analyzed with the EnVision Multilabel plate reader (Perkin Elmer). XLFit (IDBS), a Microsoft Excel Add-in, was used to analyze the data and generate dose–response curves based on standard 4- parameter logistic fit [i.e., fit = (A + (B/(1 + ((x/C)^D)))) where A and B equal minimum and maximum asymptotes, C equals IC50 and D equals slope]. The AUC values were calculated using the XLFit software utilizing minimum and maximum concentrations of drugs/compounds within the panel as the limits of the AUC calculation. For each plate, data were normalized to DMSO 100% viability and blank controls. The samples were not frozen but freshly, dissociated to single cell suspensions within hours from surgically removed tumors that were kept on ice prior to shipping.  The drug sensitivity assay was initiated within 48 hours of surgery. Clinical Laboratory Improvement Amendments standards require that thawed cell line data be repeated for standardization of the assay every 6 months. The assay results were highly reproducible.
